# Mitochondrial Neurogastrointestinal Encephalomyopathy (MNGIE-MTDPS1)

**DOI:** 10.3390/jcm7110389

**Published:** 2018-10-26

**Authors:** Massimiliano Filosto, Stefano Cotti Piccinelli, Filomena Caria, Serena Gallo Cassarino, Enrico Baldelli, Anna Galvagni, Irene Volonghi, Mauro Scarpelli, Alessandro Padovani

**Affiliations:** 1 Center for Neuromuscular Diseases, Unit of Neurology, ASST Spedali Civili and University of Brescia, 25100 Brescia, Italy; stefanocottipiccinelli@gmail.com (S.C.P.); cariafilomena@yahoo.it (F.C.); serena.gallocassarino@gmail.com (S.G.C.); enrico5589@gmail.com (E.B.); galvagni.anna@gmail.com (A.G.); irene.volonghi@hotmail.it (I.V.); alessandro.padovani@unibs.it (A.P.); 2 Department of Neuroscience, Unit of Neurology, Azienda Ospedaliera Universitaria Integrata Verona, 37100 Verona, Italy; mscarpelli1980@libero.it

**Keywords:** MNGIE, MTDPS1, mitochondrial diseases, mitochondrial therapy, mitochondrial neurogastrointestinal encephalopathy

## Abstract

Mitochondrial neurogastrointestinal encephalomyopathy (MNGIE-MTDPS1) is a devastating autosomal recessive disorder due to mutations in *TYMP*, which cause a loss of function of thymidine phosphorylase (TP), nucleoside accumulation in plasma and tissues, and mitochondrial dysfunction. The clinical picture includes progressive gastrointestinal dysmotility, cachexia, ptosis and ophthalmoparesis, peripheral neuropathy, and diffuse leukoencephalopathy, which usually lead to death in early adulthood. Other two MNGIE-type phenotypes have been described so far, which are linked to mutations in *POLG* and *RRM2B* genes. Therapeutic options are currently available in clinical practice (allogeneic hematopoietic stem cell transplantation and carrier erythrocyte entrapped thymidine phosphorylase therapy) and newer, promising therapies are expected in the near future. Since successful treatment is strictly related to early diagnosis, it is essential that clinicians be warned about the clinical features and diagnostic procedures useful to suspect diagnosis of MNGIE-MTDPS1. The aim of this review is to promote the knowledge of the disease as well as the involved mechanisms and the diagnostic processes in order to reach an early diagnosis.

## 1. Introduction

Mitochondrial Neurogastrointestinal Encephalomyopathy (MNGIE) is a rare, devastating, and progressive autosomal recessive mitochondrial disease belonging to the group of defects of inter-genomic communication associated with the depletion and multiple deletions of mitochondrial DNA (mtDNA). It is characterized by a reduction in the mtDNA copy number and the subsequent impairment of mitochondrial functions in the affected tissues [1]. Collectively named Mitochondrial DNA Depletion Syndromes (MTDPS), these diseases are clinically and genetically heterogeneous conditions caused by nuclear gene mutations disrupting deoxy ribonucleotide metabolism, which leads to an imbalance of the mitochondrial nucleotide pool and limited availability of one or more deoxy ribonucleoside triphosphates. Afterward, this results in the instability of the mitochondrial genome and the loss of mtDNA integrity [2,3]. They were numbered as MTDPS1 to MTDPS15 (Table 1).

The MNGIE type MTDPS1 (henceforth called “MNGIE”) (OMIM #603041) is caused by mutations in the *TYMP* gene located on chromosome 22q13.33, which results in the accumulation of the thymidine (dThd) and deoxyuridine (dUrd) substrates, nucleotide pool imbalance, and mtDNA instability with impairment of the mitochondrial genome replication and depletion, multiple deletions, and point mutations [4,5,6].

MNGIE is a very rare disease and its prevalence is unknown. Despite its rarity, this disease is of great interest because it is one of the few mitochondrial diseases susceptible to treatment that can potentially save the life of the patients who are otherwise condemned to a secure exitus.

The importance of a prompt recognition of symptoms and signs for a diagnosis as early as possible is, therefore, incredibly important. A late diagnosis when patients are already in poor clinical conditions greatly reduces their chances of a positive outcome after therapy.

## 2. Genetic and Biochemical Findings

*TYMP* gene encodes the thymidine phosphorylase (TP) enzyme, which is involved in the homeostasis of the mitochondrial nucleotide pool. It is a cytoplasmic enzyme expressed in most human tissues including the central and peripheral nervous system, the gastrointestinal tract, leukocytes, and platelets while it is scarcely present in muscles and is lacking in kidneys, fat tissues, and the aorta [7,8].

The TP enzyme catalyzes the first step of mitochondrial dThd and dUrd catabolism by converting them to the nucleotide bases thymine and uridine, respectively, and 2-deoxy ribose 1-phosphate [1,2,4,5,6,7,8]. As a result of TP dysfunction, MNGIE patients accumulate both dThd and dUrd in plasma and in tissues with a subsequent reduction of cytidine triphosphate (dCTP), which causes nucleoside and nucleotide pool imbalance and disrupts the equilibrium of intra-mitochondrial deoxyribonucleoside triphosphate (dNTP) pools [3,4]. dNTP imbalance interferes with mtDNA replication and accounts for the molecular alterations (mtDNA depletion, multiple deletions, and point mutations) associated with the disease [1,2,4,5].

Normal blood contains <0.05 μM of dThd and dUrd while both of them are detected at 10 to 20 μM concentrations in MNGIE patients [9,10].

*TYMP* mutations found in MNGIE patients can be homozygous or compound heterozygous mutations, are located in the exons or in their flanking regions, and range from splice site and frameshift mutations to deletions, single nucleotide insertions, and homozygous duplications [1,4,6]. Heterozygous mutation carriers have plasma nucleoside levels comparable to normal controls and, although they usually present a 35% residual TP activity, they are usually asymptomatic [7].

## 3. Clinical and Pathological Aspects

### 3.1. Classical Phenotype

Classically, MNGIE manifests as a multi-system disorder. The onset of symptoms usually occurs during the second or third decade of life and the complete clinical picture is characterized by gastrointestinal dysfunctions, cachexia, external ophthalmoparesis, eyelid ptosis, peripheral neuropathy, and leukoencephalopathy. The progressively worsening clinical course leads to death at a mean age of 37 years [4,11].

Gastrointestinal dysfunctions (HP:0002579; HP:0012850), such as bowel and gastric dysmotility, nausea, abdominal pain, distention, diarrhea, dysphagia, postprandial emesis, borborygmi, and gastroesophageal reflux are the main features of the disease [4,11,12]. This is the clinical picture of a chronic intestinal pseudo-obstruction (CIPO), which is an increasingly recognized clinical feature of mitochondrial encephalomyopathies and a highly morbid and often life-threatening condition characterized by marked dysfunction of gut propulsive motility [13].

Patients usually present with a progressive weight loss that leads to thin body habitus and severe reduction of muscle mass up to severe cachexia. They often die from their gastrointestinal disorders and an inadequate nutritional status [4,11,12].

Large small bowel diverticula at the mesenteric border, likely secondary to severe gut dysmotility, were reported in many of the studied cases and are considered suggestive of the diagnosis [11,14,15,16].

Small bowel histological examination showed features of enteric myopathy such as focal increased thickness of the inner layer of the muscularis propria, cytochrome-c-oxidase (COX) deficiency associated with mitochondrial proliferation in smooth muscle and segmentary atrophy, interstitial fibrosis, and vacuolization of smooth muscle cells in the outer layer while the large bowel appeared normal [17,18].

Electron microscopy showed many abnormally large mitochondria, rarefaction of myofibrils, and lipid accumulations in the myocytes of the muscularis propria [16,17].

In a reported case, very large mitochondria known as “megamitochondria” were detected along all the gastrointestinal tract from the esophagus to the rectum in submucosal and myenteric ganglion cells and in smooth muscle cells of muscularis mucosae and muscularis propria [19].

COX deficiency and mild decreases of mtDNA in ganglion cells in the myenteric plexus in some patients were described [17]. Evidence for interstitial cells of the Cajal (ICC) network loss around the myenteric plexus, in intermuscular septa, and within deep muscular plexus has been reported [19]. The network of the ICC has a central role in regulating gut motor function as intrinsic pacemaker cells and intermediaries of enteric neurotransmission [20]. Abnormalities of ICC presumably result in defective electrical pacemaker activity and neurotransmitter modulation with likely deleterious effects on the intestinal motor function [20].

Mitochondrial proliferation and COX deficiency were also observed in endothelial cells from the wall of small arteries and arterioles in the gastrointestinal tract and other visceral organs (liver, kidney, heart, and pancreas) [17]. This is an interesting observation, which implies a possible role for the vascular damage in MNGIE pathogenesis. Since small vessels have a key role in regulating extracellular and vascular nucleoside levels by their high-affinity nucleoside transporters, deoxynucleotide accumulation in small vessels wall (where they exert their toxic effect) could lead to endothelial cell dysfunction, which may represent a common denominator in the damage on gastrointestinal and brain tissues [17,18].

All the pathological abnormalities have been related to the presence of mtDNA depletion in the muscularis propria of the small intestine especially in its external layer as well as in small vessels. A milder reduction in mtDNA amount was observed in the stomach while the esophagus showed mtDNA deletions only in the upper esophagus and the colon did not show abnormalities [17].

Cachexia (HP:0004326) is another striking feature of MNGIE patients and it is only in part related to gastrointestinal dysfunctions [12,13,21,22]. By by-passing the dysfunctional gastrointestinal system by total parenteral nutrition (TPN), patients can recover some weight, but they remained thin despite the TPN therapy [21,22]. It implies that pathogenesis of the severe wasting is not only related to a dysfunctional intestine. By using the model systems skin and lung cultured fibroblasts, Pontarin et al. demonstrated that, in the presence of elevated concentrations of thymidine, mtDNA decreased to ~50% and the deoxythymidine triphosphate (dTTP) pool turned over rapidly due to the increased degradation and resynthesis of deoxythymidine monophosphate (dTMP) in a futile cycle between thymidine kinase and 5′-deoxyribonucleotidase [23]. The ATP-ADP turnover should be increased, which would increase the energy demand of cells. Thus, MNGIE patients could increase their consumption of ATP to counteract an unlimited expansion of the dTTP pool caused by an excess of thymidine [23].

The increased ATP consumption in the body of MNGIE patients may represent an additional toxic effect of nucleoside excess. The impaired energy balance and the subsequent increase of the body energy demand and calories consumption could play a role in the pathogenesis of the severe cachexia that MNGIE patients develop during the course of the disease.

Progressive eyelid ptosis, ophthalmoparesis, and myopathy (HP:0000508; HP:0000544; HP:0003199; HP:0002460) are a distinctive aspect of the disease [12].

Usually, patients present with a history of slowly progressive bilateral and relatively symmetrical ptosis and extra-ocular eye movement impairment. No diplopia was reported. As for other mitochondrial diseases in which a chronic ophthalmoparesis is present, i.e., Chronic Progressive External Ophthalmoplegia (cPEO) and Keyrn-Sayre Syndrome, the preferential involvement of extra-ocular muscles could be related to their specific structural and functional characteristics different from other muscle groups [24]. Their vulnerability is likely due to the high energy request with high content in mitochondria and due to dependence on oxidative phosphorylation, which make ocular muscles more prone to the effect of mitochondrial dysfunction [18].

Histological studies showed marked myopathic signs in the ocular muscles (i.e., fiber atrophy and fibrosis), which confirmed that myopathy rather than cranial neuropathy is involved in the external ophthalmoplegia [18].

A limb muscle biopsy usually shows classical signs of mitochondrial dysfunction including ragged-red fibers at Modified Gomori trichrome and scattered COX negative fibers, but it can also show only a few or no significant abnormalities [12,25]. The respiratory chain enzyme assay may show defects in oxidative phosphorylation enzyme complexes in which the most common defect is a cytochrome c oxidase (complex IV) deficiency [3,7,10]. Southern blot analysis, long-range PCR, or Multiplex ligation-dependent probe amplification (MLPA) usually show mitochondrial mtDNA major rearrangements as deletions/duplications [11]. mtDNA depletion can be observed by performing the mtDNA/nDNA ratio [4,5,6,7,8,9,10,11].

Peripheral neuropathy (HP:0007108) is a distinctive aspect of the disease and it is usually present in all the MNGIE patients [11,12,26]. It is a demyelinating neuropathy and, in some cases, it is a mixed axonal-demyelinating neuropathy. From a clinical point of you, it can be asymptomatic and pauci-symptomatic (mild sensory neuropathy) but can also present as a progressive sensory-motor neuropathy resembling a chronic inflammatory demyelinating polyneuropathy (CIDP) or as a chronic neuropathy mimicking a Charcot-Marie Tooth disease [12,27].

Distal paresthesias is a symmetrical distal weakness with a unilateral or a bilateral foot drop that may occur with a different degree of severity and different distribution in different patients [11,12,27]. Because symptoms related to the neuropathy often fluctuate in the early stages of the disease, a differential diagnosis with chronic autoimmune inflammatory neuropathies can be difficult [11,12,27].

Electroneurography (ENG) showed reduced motor and sensory nerve conduction velocities, prolonged F-wave latency, and partial conduction block. Myopathic changes on needle electromyography (EMG) are frequently found.

Nerve biopsy studies may show axonal loss especially of large myelinated fibers and onion bulbs, which indicate demyelination/remyelination processes. Mitochondrial abnormalities such as accumulation of mitochondria in Schwann cells and axons can be observed [12,27].

As in muscle tissue, mtDNA abnormalities have a non-homogeneous and segmental distribution in nerves and this can be the reason for the elettrophisiological and clinical aspect of an acquired neuropathy [28].

**Figure 1:**
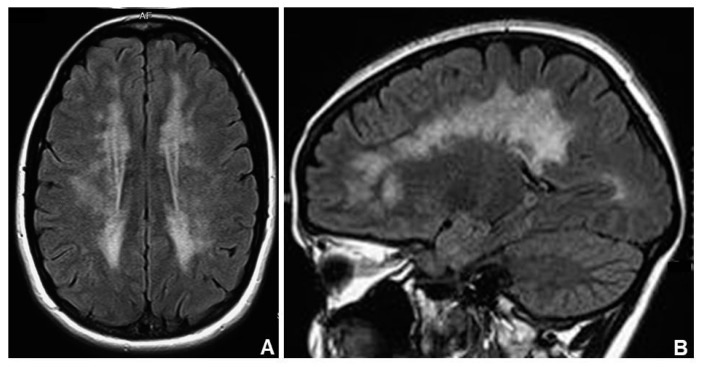
Axial (**A**) and sagittal (**B**) brain MRI (magnetic resonance imaging) view shows marked leukoencephalopathy in a MNGIE (Mitochondrial Neurogastrointestinal Encephalomyopathy) subject.

Brain leukoencephalopathy (HP:0002352) is almost always present in MNGIE patients and it is usually asymptomatic (Figure 1). The correlation between white matter involvement and central symptoms and signs, which patients can develop in the course of disease (i.e., dementia, headache, psychiatric symptoms, seizures) remains uncertain [29].

Brain magnetic resonance imaging (MRI) usually showed symmetric and confluent T2 hyper-intensities located in the cerebral white matter (with sparing of subcortical U-fibers) and, sometimes, in the cerebellar white matter in the splenium of the corpus callosum, in the basal ganglia, and in the thalami [29].

Since leukoencephalopathy is a distinctive tract of MNGIE, the brain MRI is a very useful tool in diagnosing a non-classical MNGIE phenotype. All three patients with atypical presentation (enteropathic arthritis, isolated exercise intolerance, and CIDP-like phenotype) and *TYMP* mutations had MRI findings consistent with the diagnosis of MNGIE [12].

In addition, patients with MNGIE-like phenotypes but mutations in genes different TP such as the ribonucleoside-diphosphate reductase subunit M2 B (*RRM2B*), Polymerase gamma (*POLG*), and mitochondrial DNA genes present with mild brain MRI abnormalities or no brain MRI abnormalities [30,31,32].

In our experience, the severity of phenotype and biochemical and molecular findings did not clearly correlate with the distribution and extension of the leukoencephalopathy. Thus, it should not be considered a reliable marker of disease evolution. However, other studies reported that brain MRI findings might vary with *TYMP* mutations and residual TP activity [29,33].

White matter abnormalities slowly worsen in the course of the disease regardless of a clinical course and gradually tend to become confluent and diffuse. Follow-up brain MRIs, performed at 1 and 2 years from the diagnosis in two of our patients, revealed a progression of white matter signal alterations regardless of the clinical course or treatment achieved (one patient was clinically stable without any specific therapy while a second patient showed a mild improvement after enzyme replacement therapy was begun) [29].

MR spectroscopy (MRS) represents a useful tool in studying mitochondrial diseases by evaluating an *N*-Acetyl-Aspartate (NAA) decrease (a sign of neuronal rarefaction or dysfunction), a Choline (Cho) decrease (a sign of impairment of the membrane maintenance processes due to reduced energy production), high Cho levels in the white matter (which likely reflect demyelination and membrane remodeling), and elevation of lactate, which is the end product of non-oxidative metabolism (a sign of impaired respiratory chain function) [34].

However, only a few MRS studies have been performed in MNGIE patients, which show inconsistent findings. In a study, three patients had reduced Creatine, NAA, and Cho with no lactate peaks in the white matter T2-hyperintense areas while, in a second study, no abnormalities were detected [33,35].

In three of our patients, we found NAA reduction in periventricular areas and a Cho increase in the T2-hyperintense areas of the semi-oval centers without a lactate peak. In two of these patients, MRS changes became evident in the follow-up studies when T2 hyper-intensity increased [29].

Brain pathological studies showed divergent results. In one patient, loss of myelin and reduction of the number of myelinated fibers without gliosis were found while, in two other studies, no demyelination, gliosis, or spongy degeneration were detected [36,37,38]. Pathological evaluation of the brain from an MNGIE mouse model showed multiple vacuoles in a subcortical, periventricular, internal capsule and cerebellar white matter without signs of focal demyelination [39].

An interesting post-mortem pathological evaluation showed that cortex, basal ganglia, thalamus, midbrain, and cerebellum had a uniform cytoplasmic neuronal TP staining, which is not different from normal controls while glial cells were negative for TP expression [18]. Endothelial TP staining was decreased in the white matter lesions observed on MRI ante-mortem, which indicates endothelial damage and is also described in the gastro-intestinal tract [16,18]. Pathological analysis did not show demyelination or gliosis in white matter lesions, which suggests the interesting hypothesis that the abnormal MRI signal could be related to the breakdown of the blood-brain barrier and altered blood-brain barrier permeability [29].

### 3.2. Atypical Phenotypes and Clinical Heterogeneity

Usually, the disease presents with a relatively homogeneous phenotype. However, patients with incomplete or atypical clinical manifestations have been repeatedly described and frequently misdiagnosed with conditions like anorexia nervosa, inflammatory bowel disease, CIDP, and Charcot Marie Tooth disease [12,28,40,41,42,43,44,45,46].

Some unusual symptoms have been reported in some patients such as hepatic cirrhosis with increased liver enzymes and macrovesicular steatosis, anemia, sensorineural hearing loss involving, hypergonadotropic hypogonadism, and hypogonadotropic hypogonadism [11,44,47,48].

In this regard, we observed some MNGIE patients with atypical onset symptoms as a long-standing chronic fever, recurrent acute migrant arthritis, and gastrointestinal disorders mimicking autoimmune or inflammatory intestinal diseases in one case, isolated exercise intolerance and muscle cramps in a second case, and a CIDP-like neuropathy in a third one [12].

Intra-familial phenotypical variability ranging from extremely severe to very mild clinical pictures has also been described [12,49]. To date, we followed-up a patient harboring *TYMP* mutations and with a sister who passed away from a severe form of MNGIE, which remained substantially asymptomatic seven years from diagnosis (personal observation).

No clear-cut genotype-phenotype correlation in this disease was reported but rather intra-familial and inter-familial clinical variability, which suggests a role for environmental factors and genetic modifiers in determining disease manifestations [49].

### 3.3. Other MNGIE-Type Pictures

MNGIE-like phenotypes have been described in at least two other genes involved in MTDPS.

MTDPS-4B (OMIM #613662) is an autosomal recessive multisystem disorder clinically characterized by cachexia and chronic gastrointestinal dysmotility and pseudo-obstruction associated with myopathy, progressive external ophthalmoplegia, and axonal sensory ataxic neuropathy. It is one of the so-called *POLG*-related disorders due to mutations in the *POLG* gene, which encompass a large spectrum of conditions ranging from severe cases of the Alpers-Huttenlocher syndrome to myoclonic epilepsy, myopathy, sensory ataxia (MEMSA), ataxic neuropathies, and progressive external ophthalmoplegia [31]. The muscle biopsy showed ragged-red fibers, cytochrome c oxidase negative fibers, decreased enzyme activities of respiratory chain complexes I and IV, depletion, and multiple mtDNA deletions.

MTDPS-8B (OMIM 612075) is an autosomal recessive heterogeneous condition clinically ranging from a severe early-onset encephalomyopathy with a proximal renal tubulopathy associated with mtDNA depletion to progressive external ophthalmoplegia syndromes and Kearns-Sayre syndrome linked to mtDNA deletions and MNGIE phenotypes associated with mtDNA depletion in clinically involved tissues [30].

The muscle biopsy showed increased endomysial connective tissue and ragged red fibers.

Both these disorders can be virtually, clinically indistinguishable from MNGIE due to mutations in *TYMP*. However, brain MRI is usually different and shows no leukoencephalopathy in MTDPS-4B and increased the T2-weighted signal in the basal ganglia and patchy T2-weighted signals in the periventricular and subcortical white matter in MTDPS-8B [29,30,31].

## 4. Diagnosis

The diagnosis is confirmed by a family history consistent with autosomal recessive inheritance, plasma, urine thymidine, deoxyuridine, TP enzyme activity assays, and molecular genetic testing of the *TYMP* gene [1,2,4,5].

MNGIE patients present TP enzyme activity reduced in leukocytes less than 10% of the control value and increased urine concentrations of deoxyuridine and thymidine (dThd and dUrd >3 and >5 μmol/L, respectively). Control subjects or heterozygote individuals have normal values [8,9,10,50].

A relationship between clinical phenotype and TP activity is usually accepted. Less than 10% of normal TP activity causes typical MNGIE, 10% to 20% residual activity is related to a less severe late-onset phenotypes and >30% activity does not cause evident clinical manifestations [8,9,10,50]. However, there are relevant exceptions to this rule. Late-onset or less severe phenotypes in patients with greatly reduced or virtually absent TP activity have been reported [12].

Increased serum lactate and hyperalaninemia are frequently found while lactic acidosis is rarely reported, which is more frequent if renal or hepatic impairment occurs [11].

Significantly increased protein (typically 60–100 mg/dL, normal: 15–45 mg/dL) in cerebro-spinal fluid can be observed and, especially in the variants with prominent demyelinating nerve involvement, can be misleading with a CIDP [12,28].

Differential diagnoses include, other than MNGIE-type MTDPS and CIDP, many other conditions such as anorexia nervosa, inflammatory bowel disease and irritable bowel disease, intestinal pseudo-obstruction disorders, celiac disease, and various leukodystrophies. A careful clinical evaluation is necessary to distinguish MNGIE from these different diseases [11,12,13].

## 5. Therapy

Current treatment of most mitochondrial disease remains supportive and includes vitamin cofactors, nutritional changes, and physical activity [51,52]. However, there are several exciting strategies in a development stage aimed to overcome the mitochondrial defect including strategies for enhancing mitochondrial biogenesis, removing noxious metabolites, bypassing pathogenic mechanisms, correcting biochemical defects, enhancing the respiratory chain function, scavenging free radicals, using vitamins and neuroprotective molecules, modulating aberrant calcium homeostasis, and repopulating mitochondrial DNA [51,52].

To date, the most important therapeutic advances in the field of mitochondrial diseases have been made in treating MNGIE [53].

Because systemic accumulations of dThd and dUrd are toxic, some strategies to remove the excess of nucleosides and correct the biochemical defect were studied and already introduced in clinical practice, alone or in combination, with variable results [7,52,53].

Platelets infusion to MNGIE patients partially restored TP activity and transiently reduced dThd and dUrd levels [54].

Continuous ambulatory peritoneal dialysis (CAPD) was occasionally reported as a beneficial treatment in MNGIE [55]. CAPD was performed in a patient for 22 months by using 1000 mL of 1.5% glucose dialysis fluid three times daily and 1000 mL of amino acids dialysis fluid once a day. CAPD significantly reduced plasma nucleoside levels and, after one year, obtained a clinical improvement in terms of the disappearance of vomiting, nausea, and epigastric pain, an increase in body weight, an improvement in motility and muscle strength, and an improvement of numbness. However, 15 months after the initiation of CAPD, dThd and dUrd plasma levels increased with subsequent re-appearance of gastrointestinal symptoms and severe ophthalmoplegia progression.

Hemodialysis transiently restores increased serum and urine levels of thymidine and deoxyuridine but fails to reduce CSF levels of the toxic metabolites and is ineffective to influence neurological function in a period of one year of treatment [56].

We treated a patient as a compassionate case with an enzyme replacement therapy using recombinant Escherichia coli TP entrapped in carrier erythrocyte (CEETP) [57]. In this approach, recombinant thymidine phosphorylase is encapsulated within autologous erythrocytes previously removed and subjected to reversible hypo-osmotic dialysis to enable encapsulation of TP, which are then returned to the patient. This enables the elimination of the pathological plasma metabolites. Pre-clinical studies have shown no potential serious toxicity that would preclude the clinical use of CEETP. The scientific rationale is that a sustained decrease in the systemic metabolites will arrest or reverse the progression of clinical disease. This approach has the advantage of prolonging the circulatory half-life of thymidine phosphorylase to that of the erythrocyte (19 to 29 days) and minimizing immunogenic reactions by preventing the formation of neutralizing antibodies.

Importantly, clinical assessments between 6.5 and 23 months after initiating CEETP revealed significant improvements.

This approach has been associated with a reduction of plasma and urine thymidine and deoxyuridine. Although periodical infusions are needed, CEETP should be considered a rescue or maintenance therapy for MNGIE patients prior to the availability of a suitable allogeneic hematopoietic stem cell transplantation or liver donor or as an alternative therapy for patients who have irreversible end-stage disease and are without an optimally matched donor.

Allogeneic hematopoietic stem cell transplantation (HSCT) is a well-defined treatment option for MNGIE [58,59,60,61]. This approach, however, has serious limitations including the difficulty in obtaining suitable donors, the toxicity of the conditioning regimen, and the risk of graft failure and graft vs. host disease. In addition, MNGIE patients are generally in a poor medical condition at the time of the diagnosis and the treatment can be associated with high morbidity and mortality rates.

A consensus conference proposal for a standardized approach to HSCT in MNGIE suggested that, if no sibling donor can be found, an HSCT with a 10/10 allele matched an unrelated donor (human leucocyte antigen system (HLA)-A, B, C, *DRB1*, and *DQB1* phenotypically identical) is recommended [60]. A single mismatch at HLA-A, -B, -C, or -DRB1 has been associated with about a 9% increase in the mortality risk [58,59,60].

We treated two MNGIE patients with HSCT [59]. The source of stem cells was bone marrow taken from an HLA 9/10 allele-matched unrelated donor in the first patient and from an HLA 10/10 allele-matched sibling donor in the second. Both patients achieved full donor chimerism and we observed restoration of buffy coat TP activity and lowered urine nucleoside concentrations in both of them. The post-transplant clinical follow-up showed improvement in gastrointestinal dysmotility, abdominal cramps, and diarrhea. The neurological assessment remained unchanged.

However, the first patient died 15 months after HSCT due to gastrointestinal obstruction and shock. The second patient died 8 months after the procedure due to respiratory distress following septic shock.

A retrospective analysis of all known patients suffering from MNGIE treated with allogeneic hematopoietic stem cell transplantation between 2005 and 2011 showed that 9 of 24 patients (37.5%) were alive during the last follow-up with a median follow-up of surviving patients after 1430 days [58]. Seven patients (29%) living more than two years after transplantation presented improvement of gastrointestinal manifestations and peripheral neuropathy and an increase in the body mass index [58]. Complications linked to transplantation caused deaths in nine patients while MNGIE progression was considered the cause of death in six patients [58].

The human leukocyte antigen match (10/10 vs. <10/10) and disease characteristics (liver disease, history of gastrointestinal pseudo-obstruction, or both) were two findings strongly associated with survival [58].

Available data confirm that HSCT restores the thymidine phosphorylase enzyme function and improves the clinical picture. However, due to a high complication rate, it should be considered for selected patients with an optimal donor and optimal clinical conditions when they are still relatively healthy [39,58,62].

Nevertheless, the rule of “early transplantation” is difficult to routinely apply in MNGIE patients because a frequent misdiagnosis causes a delay in achieving the correct diagnosis and the incomplete knowledge about the natural history in many affected patients [59].

The use of autologous hematopoietic stem cells genetically engineered to produce normal *TYMP* would eliminate many of the risks linked to allogeneic HSCT and also has the intrinsic advantage of inducing immune tolerance to the recombinant therapeutic transgene product [63,64]. TP-deficient B-lymphoblastoid cells from two MNGIE patients were transduced with lenti-viral vectors carrying a functional copy of the human *TYMP* DNA coding sequence. This restored TP activity in the cells, which reduced the excretion of dThd and dUrd and their concentrations when added in excess. Lentiviral-mediated hematopoietic gene therapy was used in partially myeloablated double *Tymp*/*Upp1* knockout mice and, again, high levels of TP activity were observed in the peripheral blood of the transplanted mice with a concomitant reduction of nucleoside concentrations. These promising findings suggest that hematopoietic gene therapy could be an interesting alternative treatment for MNGIE in the future.

More recently, it has been reported that treatment with AAV2/8-mediated transfer of the human *TYMP* coding sequence (hcTYP) targeting the liver in a murine model provides a permanent biochemical correction without adverse effects, which further indicates that gene therapy is a feasible therapeutic option for MNGIE treatment [65].

Liver transplantation may represent a suitable alternative option for treating patients with MNGIE. Since there is a high thymidine phosphorylase expression in the liver and transplantation success is estimated at 90% of cases, it has been proposed as a good source of TP [66]. A 25-year-old severely affected MNGIE patient underwent liver transplantation. Serum levels of toxic nucleosides rapidly normalized. At 400 days of follow-up, the patient’s clinical conditions are stable.

## 6. Conclusions

MNGIE summarizes the main characteristics of mitochondrial diseases: clinical variability, multisystem involvement, and a severe outcome.

However, unlike treatment for most mitochondrial diseases, potentially disease modifying therapies are available for MNGIE. Although with obvious limitations, i.e., high mortality rate for HSCT, transient effect for CEETP, low experience with liver transplantation, MNGIE is a significant example of translational medicine. It is a way through which research studies reveal a specific rationale for treatment.

If the task of the clinicians is early diagnosis, the task of scientific research is improving and optimizing the current therapeutic options and finding new ones and possibly expanding this knowledge to other fields of mitochondrial medicine.

## Figures and Tables

**Table 1:** Mitochondrial DNA Depletion Syndromes divided according to the phenotypes (modified from [3]). A and B indicate different phenotypes linked to the same gene.

Phenotype	Gene	Number	Other Clinical Findings
Hepatocerebral	*DGUOK*	3	
	*POLG*	4A	Alpers type
	*MPV17*	6	
	*TWNK* (*C10orf2*)	7	
	*TFAM*	15	
Encephalo-myopathic	*SUCLA2*	5	associated with methylmalonic aciduria
	*FBXL4*	13	
	*SUCLG1*	9	associated with methylmalonic aciduria
	*RRM2B*	8A	associated with renal tubulopathy
	*OPA1*	14	encephalocardiomyopathic type
Neurogastro-intestinal	*TYMP*	1	MNGIE type
	*POLG*	4B	MNGIE type
	*RRM2B*	8B	MNGIE type
Myopathic	*TK2*	2	
	*AGK*	10	cardiomyopathic type
	*MGME1*	11	
	*SLC25A4*	12A	autosomal dominant cardiomyopathic type
	*SLC25A4*	12B	autosomal recessive cardiomyopathic type
